# Adaptations for extremely high muscular power output: why do muscles that operate at intermediate cycle frequencies generate the highest powers?

**DOI:** 10.1007/s10974-022-09640-2

**Published:** 2023-01-11

**Authors:** Graham N. Askew

**Affiliations:** School of Biomedical Sciences, University of Leeds, LS2 9JT Leeds, England

## Abstract

The pectoralis muscles of the blue-breasted quail *Coturnix chinensis* generate the highest power output over a contraction cycle measured to date, approximately 400 W kg^− 1^. The power generated during a cyclical contraction is the product of work and cycle frequency (or standard operating frequency), suggesting that high powers should be favoured by operating at high cycle frequencies. Yet the quail muscles operate at an intermediate cycle frequency (23 Hz), which is much lower than the highest frequency skeletal muscles are capable of operating (~ 200 Hz in vertebrates). To understand this apparent anomaly, in this paper I consider the adaptations that favour high mechanical power as well as the trade-offs that occur between force and muscle operating frequency that limit power. It will be shown that adaptations that favour rapid cyclical contractions compromise force generation; consequently, maximum power increases with cycle frequency to approximately 15–25 Hz, but decreases at higher cycle frequencies. At high cycle frequencies, muscle stress is reduced by a decrease in the crossbridge duty cycle and an increase in the proportion of the muscle occupied by non-contractile elements such as sarcoplasmic reticulum and mitochondria. Muscles adapted to generate high powers, such as the pectoralis muscle of blue-breasted quail, exhibit: (i) intermediate contraction kinetics; (ii) a high relative myofibrillar volume; and (iii) a high maximum shortening velocity and a relatively flat force-velocity relationship. They are also characterised by (iv) operating at an intermediate cycle frequency; (v) utilisation of asymmetrical length trajectories, with a high proportion of the cycle spent shortening; and, finally, (vi) relatively large muscles. In part, the high power output of the blue-breasted quail pectoralis muscle can be attributed to its body size and the intermediate wing beat frequency required to generate aerodynamic force to support body mass, but in addition specialisations in the contractile and morphological properties of the muscle favour the generation of high stress at high strain rates.

**Supplementary information:**

The online version contains supplementary material available at 10.1007/s10974-022-09640-2.

## Introduction

Many activities that animals perform require a high mechanical power output, often those associated with escaping from predators or capturing prey where the very survival of an animal may depend on the power generated. In animal locomotory systems, muscle is ultimately the source of the power, which is generated during the crossbridge cycle. Instantaneous power, the product of force and shortening velocity, is determined by the intrinsic contractile properties of the muscle. The highest instantaneous and cycle average power outputs of skeletal muscle measured to date are approximately 1200 W kg^− 1^ and 400 W kg^− 1^, respectively, in the pectoralis muscles of the blue-breasted quail *Coturnix chinensis* (Askew and Marsh 2001, 2002), a muscle that operates at an intermediate cycle frequency of 23 Hz. Why is the most powerful muscle one that operates at an intermediate cycle frequency and what limits power in muscles that operate at cycle frequencies above or below these intermediate frequencies? The goal of this review is to analyse published data on muscle performance to reveal what limits power generation at different cycle frequencies and to understand the adaptations that favour high direct power generation.

## Scaling of maximum net muscle power output with cycle frequency

**Fig. 1:**
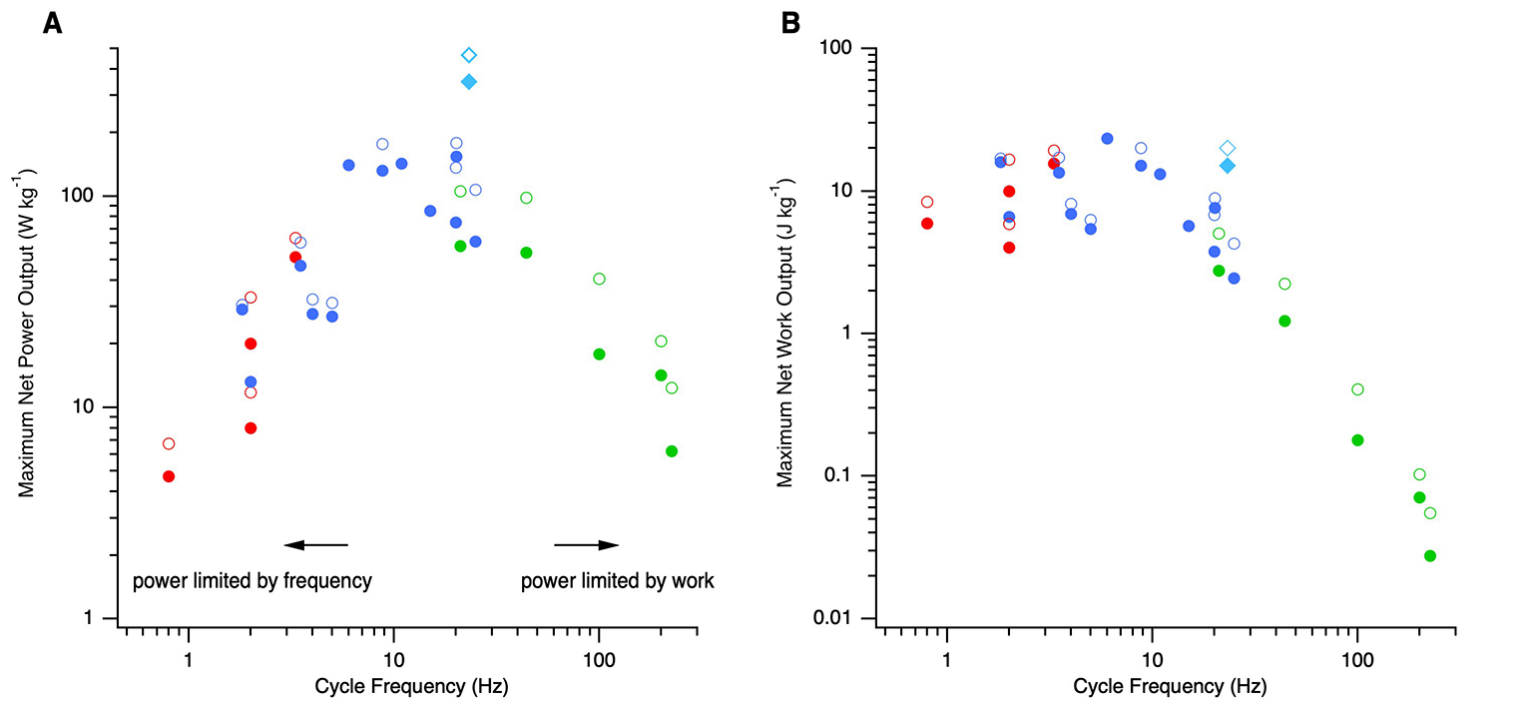
Relationship between (A) muscle mass-specific maximum net power and (B) muscle mass-specific work and cycle frequency. Solid symbols indicate mass-specific whole muscle power and open symbols mass-specific myofibrillar power, with red and blue indicating slow and fast locomotory muscles and green vocalisation muscles, respectively; the blue-breasted quail pectoralis muscle (a fast locomotory muscle) is indicated by the turquoise diamond

Theoretically, based on assumptions about the geometric similarity of animals and the contractile properties of their muscles, maximum muscle power output is predicted to scale in proportion with cycle frequency (or standard muscle operating frequency) with work per cycle being constant (Hill 1950; Pennycuick and Rezende 1984). The relationship between maximum net mass-specific power (in W kg^− 1^; hereafter power) and cycle frequency for a range of muscles that operate at a range of frequencies in vivo, is illustrated in Fig. 1 A. This is a subset of published data, selected because additional data on contraction kinetics and muscle composition are available, that will give insight into the adaptations for a high power output (data and references presented in the Supplementary Information). Maximum power increases with cycle frequency, as predicted by theory, but only up to cycle frequencies of approximately 15–25 Hz; above these frequencies power decreases (Fig. 1 A), indicating that some of the assumptions underlying the theoretical prediction (noted by Marsh 1988; Marsh 1994), do not hold across all cycle frequencies. Cyclical net mass-specific work (in J kg^− 1^; hereafter work) is not constant but decreases at cycle frequencies above ~ 20–25 Hz (Fig. 1B).

Muscles that are adapted to operate at intermediate cycle frequencies in the range 15–25 Hz, such as the pectoralis muscles of blue-breasted quail (Askew and Marsh 2001), generate the highest maximum power output. At cycle frequencies below ~ 15 Hz, power is limited by cycle frequency, whereas above ~ 25 Hz, power is limited by work (Fig. 1). To understand why muscles operating in this frequency range generate the highest powers, it is necessary to consider the specific adaptations that determine those physiological properties that influence power – namely adaptations that maximise force and shortening velocity – and their relationship with muscle operating frequency.

## Skeletal muscle adaptations for high power output

The force and shortening velocity of a muscle are, in part, determined by the overall size of the muscle, and therefore one adaptation to increase absolute muscular power, is simply to increase the relative size of the muscles driving the activity. Muscle hypertrophy can occur as the result of exercise training (Schoenfeld 2010), androgenic steroids (Bhasin et al. 2001) or on an evolutionary timescale. For example, the size of the vocalisation muscles of male hylid tree frogs varies seasonally, increasing during the breeding season approximately two-fold when plasma testosterone levels are higher (Girgenrath and Marsh 2003). Many predominantly ground-dwelling birds such as those in the pheasant family perform explosive take-off flights as a means of escape and these species have relatively large pectoralis muscles (Askew and Marsh 2002) with an origin on a relatively larger and elongated sternum (Lowi-Merri et al. 2021) compared to other families that do not perform such flights. However, while the overall size of the muscle is important in determining the absolute power generated by the animal, which will determine its performance, power normalised to muscle mass highlights the effects of other physiological properties of the muscle. Therefore, in addition to differences in the relative size of muscles, it is the intrinsic force generating capacity and force-velocity characteristics of the muscle that determine the muscle’s power output.

**Fig. 2:**
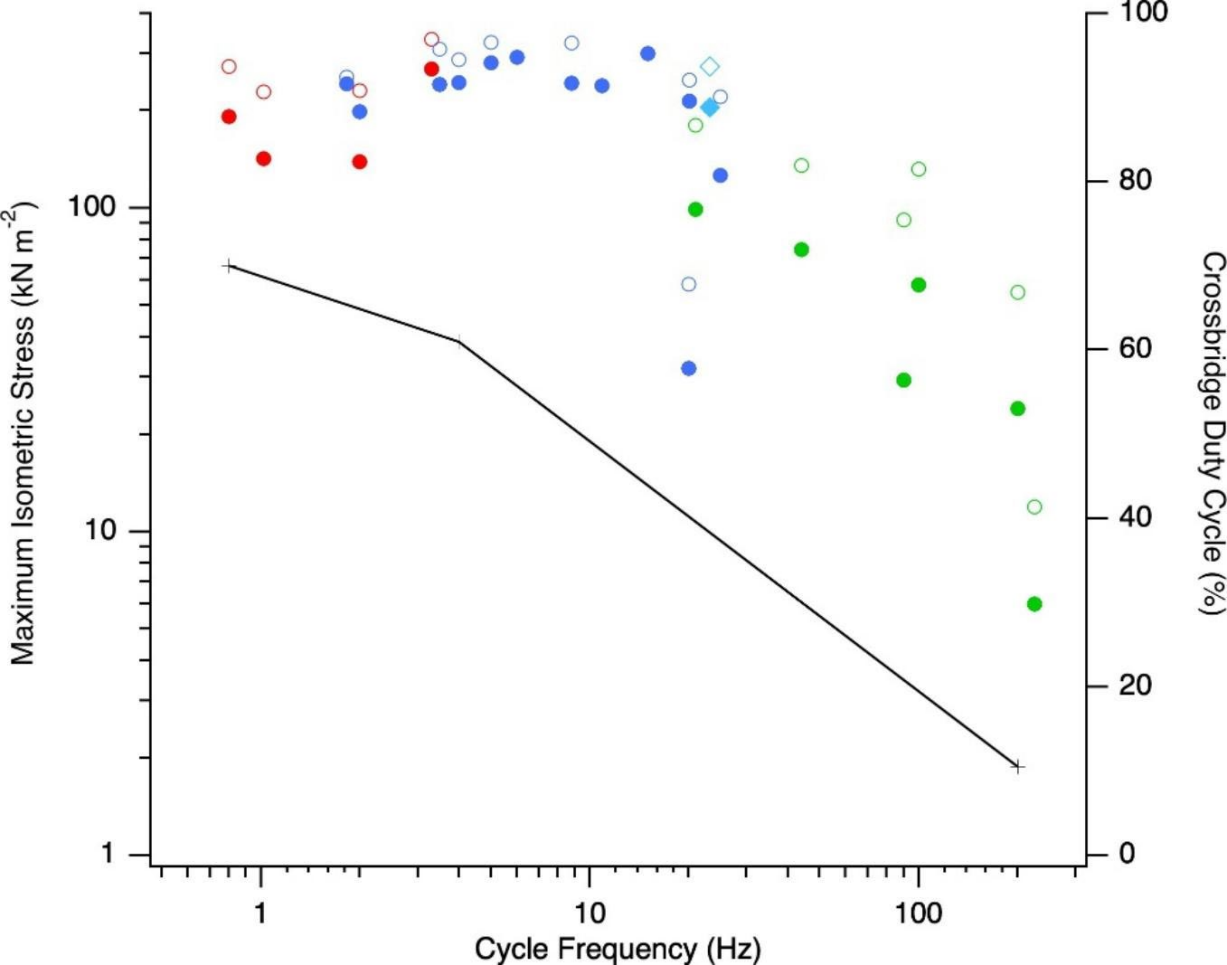
Variation in maximum isometric stress with cycle frequency. Solid symbols indicate whole muscle isometric stress and open symbols myofibrillar isometric stress, with red and blue indicating slow and fast locomotory muscles and green vocalisation muscles, respectively; the blue-breasted quail pectoralis muscle (a fast locomotory muscle) is indicated by the turquoise diamond. Crosses indicate the crossbridge duty cycle in toadfish red, white and swimbaldder muscle

### Adaptations that increase muscle force

The isometric tetanic myofibrillar stress – that is the maximum isometric force that can be generated per unit myofibrillar cross-sectional area – is often considered to be relatively constant in vertebrate skeletal muscles; ~250–400 kN m^− 2^ (Weis-Fogh and Alexander 1977). In vertebrates, the uniformity in the spacing between the thick and thin filament lattice in the sarcomeres and the conserved dimensions of the thick filaments, results in a constant maximum number of crossbridge heads that can form (i.e. the number that would be attached during rigor). Many locomotory muscles do have a maximum isometric myofibrillar stress that falls within this 250–400 kN m^− 2^ range; however, there are examples of muscles that generate much lower stresses (Fig. 2). During an isometric contraction, not all of the crossbridges are in a strongly attached force-generating state throughout the entire crossbridge cycle. In frog tibialis anterior muscle, it has been estimated that no more than 43% of crossbridges of the crossbridges that are attached during rigor are attached during an isometric contraction (Linari et al. 1998). The proportion of crossbridges attached (termed the crossbridge *duty cycle*) during an isometric contraction differs between muscles, and is related to the cycle frequency at which the muscle operates *in vivo* (Rome et al. 1999). Crossbridge duty cycle is lower in muscles that operate at higher cycle frequencies, leading to some variation in the maximum isometric myofibrillar stress generated in muscles that operate at different cycle frequencies. For example, in the toadfish the proportion of attached crossbridges is 70% in red muscle (estimated operating frequency 0.8 Hz), 61% in white muscle (operating frequency 4 Hz) and 10.5% in swimbladder muscle (operating frequency 200 Hz), with corresponding myofibrillar stresses of approximately 280 kN m^− 2^ in the locomotory (red and white) muscles but only 55 kN m^− 2^ in the swimbladder muscle (Rome et al. 1999). Low myofibrillar stresses (12–92 kN m^− 2^) stresses appear to be a feature of muscles involved in vocalisations that are specialised to operate at high cycle frequencies (90–225 Hz) such as the toadfish swimbladder muscle (Rome et al. 1999), rattlesnake shaker muscle (Martin and Bagby 1981; Moon et al. 2002) and avian syringeal muscles (Elemans et al. 2004; Adam et al. 2021). The low myofibrillar stresses arise as a result of these muscles having relatively high crossbridge detachment rates compared to their crossbridge attachment rate, an adaptation that is necessary to enable contraction and relaxation to occur within the brief period of the cycle, and that results in relatively few attached crossbridges during contraction (Rome et al. 1999). The high crossbridge detachment rate and the low myofibrillar stresses in muscles that operate at high cycle frequencies, is likely related to the unique myosin heavy chain genes expressed (Mead et al. 2017). Thus, in terms of power generation, there is a trade-off between muscle operating frequency and force generation in muscles operating at cycle frequencies above ~ 25 Hz; above this frequency, net work per cycle decreases (Fig. 1B). However, below this frequency myofibrillar stress (and work) is relatively constant. Above cycle frequencies of ~ 25 Hz, power will be compromised if the decrease in the stress generated during shortening is greater than the increase in strain rate.

In addition to variation in myofibrillar stress related to muscle operating frequency, there is also variation in both intra- and inter-specific muscle stress (Fig. 2). Beyond differences resulting from variation in myofibrillar stress, muscle fibre type is a key determinant of muscle stress. The red and white locomotory muscles in toadfish have very similar myofibrillar stresses (~ 280 kN m^− 2^) but the white muscle has a stress that is 27% higher than the red muscle (Rome et al. 1999). The main reason for the difference in muscle stress is the presence of different proportions of non-contractile elements in different muscle fibre types. For example, fast glycolytic fibres have a high proportion of their cross-sectional area made up of myofibrils (80–90%) and in these muscle fibre types, stress is typically comparable to that of the myofibrils (e.g. muscle stress in frog hindlimb muscles is 240–285 kN m^− 2^; McLister et al. 1995, Peplowski and Marsh 1997). However, the cross-sectional area of muscle fibre types reliant on aerobic metabolism such as slow and fast oxidative glycolytic fibres and/or operate at relatively high cycle frequencies, includes a proportion of non-contractile elements such as mitochondria, sarcoplasmic reticulum, capillaries and fuel reserves (lipid, glycogen) which reduces the muscle stress below that of the myofibrils themselves. For example, the external oblique muscles that power vocalisation in gray tree frogs contain approximately 20% mitochondria, 14% lipid droplets and 55% myofibrils and have an isometric muscle stress of 85–104 kN m^− 2^ somewhat lower than the stress generated by the locomotory muscles of the same species (231 kN m^− 2^), which comprise 6% mitochondria, 2% lipid droplets and 84% myofibrils (Marsh and Taigen 1987; Girgenrath and Marsh 1999; Marsh 1999).

### Force-velocity properties

**Fig. 3:**
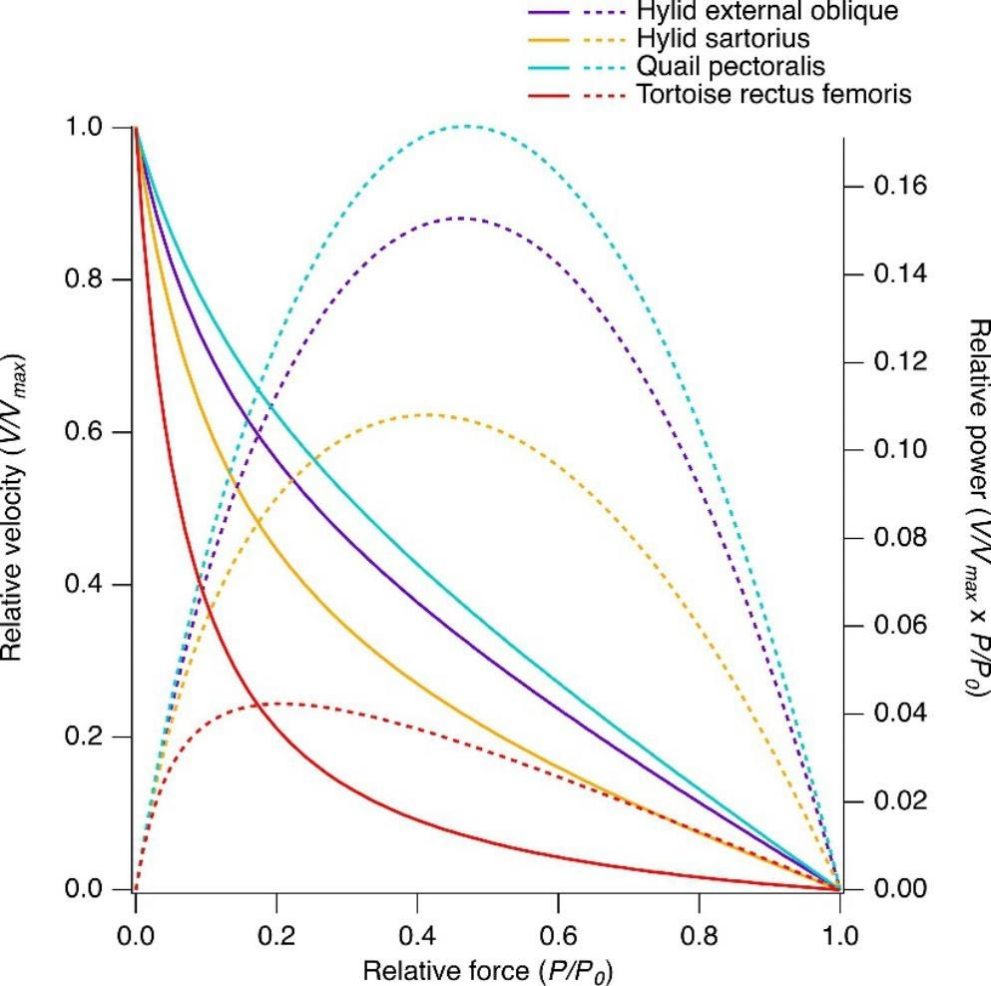
The effects of the curvature of the force-velocity relationship on relative power. Force (*P*) and velocity (*V*) are plotted relative to maximum isometric force (*P*_0_) and maximum shortening velocity (*V*_max_), respectively. Data are for hylid external oblique and sartorius muscles, blue-breasted quail pectoralis muscle and tortoise rectus femoris muscle, which span the range of known curvatures or power ratios (Marsh 1999, G.N. Askew and R.L. Marsh, unpublished data, Woledge 1968). Solid lines represent the force-velocity relationship and dashed lines the power-force relationship

The inverse relationship between force and velocity yields a power output of zero at *V*_max_ and under isometric conditions where force and velocity are zero, respectively, and power is maximal at an intermediate relative velocity where the muscle generates an intermediate relative force (Fig. 3; Rome et al. 1988). Differences in crossbridge detachment rate result in an approximately 80-fold variation (Medler 2002; Woledge et al. 1985; Askew and Marsh 2001) in the maximum velocity of shortening (*V*_max_) in skeletal muscle, with a higher *V*_max_ favouring a higher power output.

The relative force and velocity at which power is maximal depends on the curvature of the force-velocity relationship. Muscles with a relatively flat force-velocity relationship generate power at relatively higher shortening velocities compared to those with more curved force-velocity relationships and generate higher relative power (Fig. 3). The power ratio indicates the curvature of the force-velocity relationship (calculated by dividing the peak power by the product of *V*_max_ and *P*_0_; Marsh and Bennett 1986) and ranges from 0.042 in the highly curved force-velocity relationship of tortoise muscle (Woledge 1968) to 0.15–0.17 in the vocalisation muscles of tree frogs (Marsh 1999) and pectoralis muscle of quail (G.N. Askew and R.L. Marsh, unpublished data). The low-curvature force-velocity relationships in the external oblique muscles of tree frogs and blue-breasted quail pectoralis develop maximum power at 0.44–0.47 *P*_0_ and 0.36–0.38 *V*_max_ (Girgenrath and Marsh 1999; G.N. Askew and R.L. Marsh, unpublished data; Fig. 3) and develop higher relative power than locomotory muscles with more curved force-velocity relationships (Marsh 1999). Therefore, muscles specialised for generating high power, such as the blue-breasted quail pectoralis muscle, have a high *V*_max_ and a relatively flat force-velocity relationship.

### Contraction kinetics and power generation during cyclical contractions

**Fig. 4:**
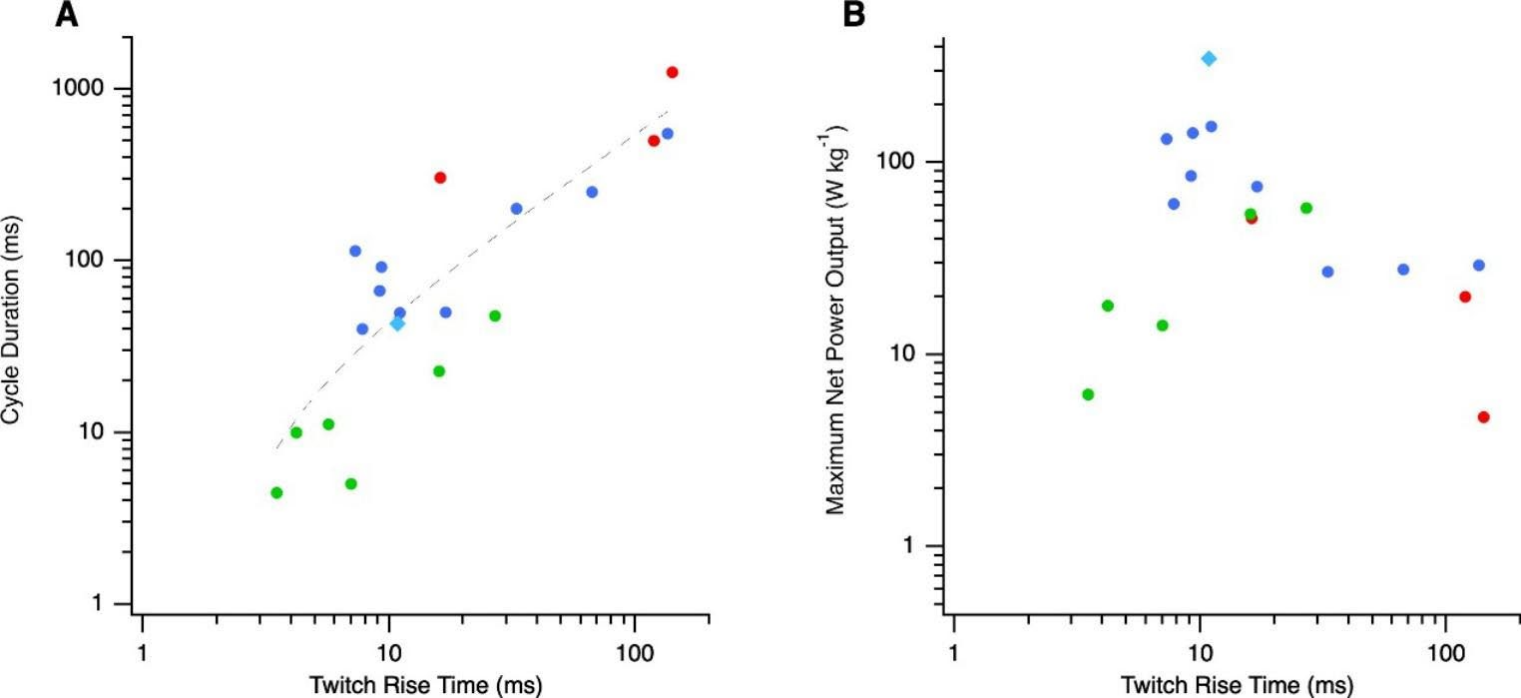
The effects of twitch rise time on (A) cycle duration and (B) maximum net power output. Red and blue symbols indicate slow and fast locomotory muscles and green vocalisation muscles, respectively; the blue-breasted quail muscle (a fast locomotory muscle) is indicated by the turquoise diamond. In panel A, the dashed line is the linear regression of cycle duration against twitch rise time

The force-velocity relationship and maximum isometric force largely determine a muscle’s maximum instantaneous power output. Some ballistic movements are driven by a single muscle contraction and the maximum instantaneous power output is the main determinant of performance. However, many muscles undergo repetitive oscillatory length changes during which there are distinct power and recovery phases of the cycle. In this type of activity, there are additional factors that influence power. In order to maximise net power output, the muscle must develop active force predominantly during muscle shortening and relax during the recovery phase while the muscle is re-lengthened. Maximal net power during a cyclical contraction would be generated if the muscle developed force and relaxed instantaneously, however, the processes of excitation contraction coupling and sequestration of calcium ions take a finite amount of time. The time taken for force to rise means that the optimal phase of activation of the muscle for maximum power output occurs during lengthening, increasing the work done on the muscle to stretch it. Also, the time taken for the muscle to relax means that muscle activation must cease during shortening to enable time for force to decline before being re-lengthened. Submaximal activation reduces the work generated during shortening and force generated during lengthening increases the work that must be done re-lengthening the muscle during, compared to that which could be generated if the muscle could contract and relax instantaneously. Rapid contraction kinetics minimise this reduction in net work. Adaptations for rapid Ca^2+^ transients, result in rapid contraction kinetics. Rapid release of Ca^2+^ into the sarcoplasm is favoured by an increased density of dihydropyridine receptors in the transverse tubular system, increased concentration of ryanodine receptors (the Ca^2+^ release channels) either due to an increased density in the sarcoplasmic reticulum (SR) membrane or an increased relative volume of SR in the muscle (Appelt et al. 1991; Baylor and Hollingworth 2003), and is more rapid in fast muscle compared to slow. However, differences in the rate of Ca^2+^ release are not thought to limit muscle operating frequency (Syme and Josephson 2002). It is the rate at which sarcoplasmic Ca^2+^ concentration falls that determines the rate at which Ca^2+^ dissociates from troponin, the contraction kinetics and the cycle frequency limit; hence the linear relationship between contraction kinetics and cycle duration (Fig. 4 A). An increased sarcoplasmic reticulum pump density in the SR membrane and rapid binding and release of calcium from troponin, are adaptations for rapid contraction kinetics enabling muscles to operate at high cycle frequencies (Rome and Lindsted 1998; Baylor and Hollingworth 2003). Additionally, some fast-muscles have a high concentration of parvalbumin – a muscle protein that binds myoplasmic calcium ions, facilitating rapid relaxation (Rome 2006). However, there is clearly a trade-off between having rapid calcium transients and generating high maximum isometric muscle force, due to the reduction in relative myofibrillar volume and consequently the reduction in muscle stress, that occurs as the relative volume of SR increases. This trade-off means that muscles that operate at very high cycle frequencies, requiring rapid twitch kinetics, do not generate very high powers (e.g. see high-frequency vocalisation muscles in Fig. 4B). Therefore, muscles that are adapted to generate high power are expected to have rapid twitch kinetics, but not at the extreme range for skeletal muscle – muscles with twitch times below approximately 8 ms and above 30 ms appear to have compromised power, compared to muscles with twitch times that fall within this range (Fig. 4B).

### Muscle length trajectory

**Fig. 5:**
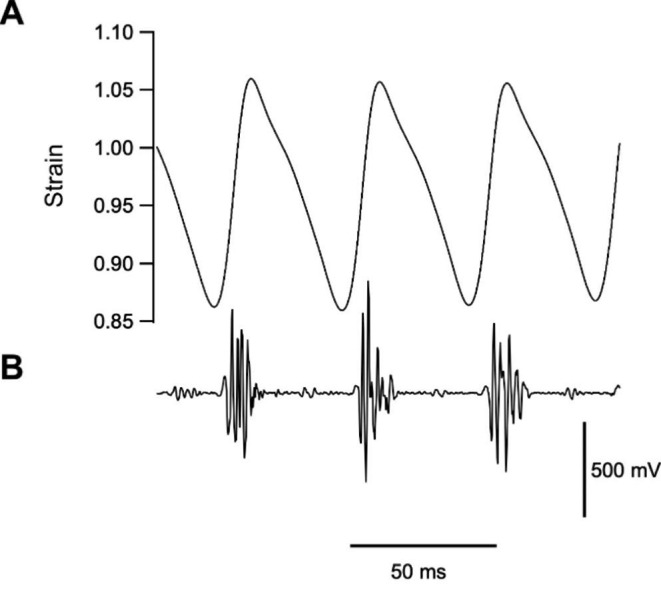
*In vivo* muscle length change and activity pattern in the pectoralis muscle of blue-breasted quail *Coturnix chinensis* during take-off flight. **A** Muscle length change (determined using sonomicrometry) relative to the resting length of the muscle prior to the flight. The proportion of the cycle spent shortening is 70%, strain is 23% and cycle frequency is 23 Hz. **B** muscle activity (determined using electromyography). Data from Askew and Marsh 2001

In addition to rapid contraction kinetics, net power during cyclical contractions can also be increased by operating with asymmetrical length trajectories in which the proportion of the cycle spent shortening is higher than that spent lengthening. Such asymmetrical cycles have been observed in many power generating muscles, including the pectoralis muscle of many birds that perform explosive take-off flights (e.g. 70% of the cycle is spent shortening in blue-breasted quail pectoralis muscle; Fig. 5; Askew and Marsh 2001), in the pectoralis muscles of birds flying at speeds where the power requirements are highest (e.g. 69% of the cycle is spent shortening at slow flight speeds compared to 56% at intermediate flight speeds in the pectoralis muscle of budgerigar; Ellerby and Askew 2007), and in frog vocalisation muscles (e.g. 65–75% of the cycle is spent shortening in the gray tree frog; Girgenrath and Marsh 1997). Why do asymmetrical length trajectories lead to increased net power output? Firstly, the increase in the time spent shortening allows the muscle to achieve a greater level of activation during shortening, which may enable the duration of activation to be increased, resulting in a higher mean force over the cycle (Askew and Marsh 1998). Secondly, the optimal relative shortening velocity decreases as the time spent shortening increases (Askew and Marsh 1998). This increases the optimal strain and the muscle generates a higher relative force due to the force-velocity relationship (the muscle shortens at lower velocities). Thirdly, the relatively shorter period of lengthening increases the rate of activation, meaning that force rises more quickly, reducing the amount of work that needs to be done on the muscle during lengthening and resulting in a greater level of activation during shortening (Askew and Marsh 1998). Therefore, to generate high power outputs, muscles should operate with asymmetrical length trajectories with a greater proportion of the cycle spent shortening than lengthening.

### Conclusions: muscle designed for maximum cyclical power

There is a trade-off between force generation and the frequency at which the muscle operates, such that the highest powers are generated in muscles that operate at intermediate cycle frequencies. Muscles that generate high power exhibit: (i) intermediate contraction kinetics; (ii) a high relative myofibrillar volume; and (iii) a high maximum shortening velocity and a relatively flat force-velocity relationship. They are also characterised by (iv) operating at an intermediate cycle frequency; (v) utilising an asymmetrical length trajectory, with a high proportion of the cycle spent shortening; and, finally, (vi) relatively large muscles.

The highest power output over a contraction cycle yet measured is ~ 400 W kg^− 1^ in the pectoralis muscle of blue-breasted quail, which powers explosive, escape take-off flights and which operates at an *intermediate cycle frequency* of 23 Hz (Askew and Marsh 2001, 2002; Askew et al. 2001). The wingbeat frequency of the blue-breasted quail (23 Hz; Askew et al. 2001), is determined by the animal’s body mass, being dependent on wing size and the aerodynamic force needed to support body mass (Ellington 1991). The requirement to operate at 23 Hz, has resulted in *intermediate contraction kinetics*, with a twitch rise time of 10.8 ms and twitch half-relaxation of 8.8 ms (Askew and Marsh 2001). Intermediate contraction kinetics appear to be optimal for maximising power (Fig. 4) by avoiding the reduction in muscle stress and work that results from the increase in the relative non-contractile fraction of the muscle and reduction in crossbridge duty cycle associated with operating at higher cycle frequencies (Figs. 1, 2 and 3). Consequently, the high power output of the pectoralis muscle of blue-breasted quail can be attributed, in part, to its body mass.

The blue-breasted quail pectoralis muscle has a *high relative myofibrillar volume* due to the predominantly type IIb fibre composition (67% type IIb, 33% type IIa with a muscle myofibrillar volume of 75%; Boesiger 1992; Kiessling, 1977; Rosser et al., 1987; Electronic Supplementary Information), resulting in a relatively high muscle stress (Johnston 1985). The relative importance of possessing a high myofibrillar volume in yielding a high stress can be appreciated by comparing the stress generated by the blue-breasted quail pectoralis muscle to the external oblique muscles of the gray tree frog *Hyla versicolor*, which operates at a similar cycle frequency (21 Hz; Girgenrath and Marsh 1997), but is composed of type IIa fibres, with a lower proportion of myofibrils (55%) and relatively high content of non-contractile components (Marsh and Taigen 1987): the muscle stress in blue-breasted quail pectoralis muscle is approximately 2× that of *H. versicolor* external oblique muscles.

The blue breasted quail pectoralis muscle has a very *low curvature force-velocity relationship*, resulting in a high relative power (Fig. 3; measured at 40 °C, G.N. Askew and R.L. Marsh, unpublished data). The *H. versicolor* external oblique muscles also have relatively flat force-velocity relationships, with a relative peak power 76% (Marsh 1999) that of blue breasted quail pectoralis muscles. However, mass-specific power depends also on the maximum isometric stress and the maximum shortening velocity of the muscle. The *high maximum shortening velocity* of the blue breasted quail pectoralis muscle (32 *L* s^− 1^; measured at 40 °C, G.N. Askew and R.L. Marsh, unpublished data), and high muscle stress result in a peak mass-specific isotonic power that is 9.5× that generated by the hylid external oblique muscles. Compared to the highly curved force-velocity relationship of tortoise rectus femoris and extremely low maximum shortening velocity (Woledge 1968), mass-specific isotonic power of blue-breasted quail pectoralis muscle is 24× higher.

During explosive take-off flights, the pectoralis muscle exhibits an *asymmetrical length trajectory*, with 70% of the cycle spent shortening (Askew and Marsh 2001). This asymmetrical length trajectory, together with the force-velocity properties of the muscle (high *V*_max_ and flat force-velocity relationship), allows the muscle to undergo a relatively large strain, shorten at a high strain rate (7.8 muscle lengths s^− 1^) and develop a relatively high mean stress (Askew and Marsh 2002). The relative importance of utilising asymmetrical length trajectories is demonstrated by the 1.4 to 1.6× more power that can be generated during asymmetrical cycles (60 to 75% of the cycle spent shortening) than in symmetrical cycles (Askew and Marsh, 1997; Girgenrath and Marsh 1999).

Adaptations that enable high power generation may compromise other aspects of animal performance. Generation of high work and reliance on anaerobic metabolism results in muscles that cannot sustain power as they rapidly fatigue. In blue-breasted quail explosive flights are the initial, rapid-response, anti-predation behaviour of these birds, that are followed by escape by sustained running.

## Electronic supplementary material

Below is the link to the electronic supplementary material.

Supplementary Material 1

Supplementary Material 2

## References

[CR1] Adam I, Maxwell A, Rossler H, Hansen EB, Vellema M, Brewer J, Elemans CPH (2021). One-to-one innervation of vocal muscles allows precise control of birdsong. Curr Biol.

[CR2] Appelt D, Shen V, Franzini-Armstrong C (1991). Quantification of ca ATPase, feet and mitochondria in superfast muscle fibers from the toadfish, Opsanus tau. J Muscle Res Cell Motil.

[CR3] Askew GN, Marsh RL (1998). Optimal shortening velocity (*V/V*_max_) of skeletal muscle during cyclical contractions: length–force effects and velocity-dependent activation and deactivation. J Exp Biol.

[CR4] Askew GN, Marsh RL (2001). The mechanical power output of the pectoralis muscle of blue-breasted quail (Coturnix chinensis): the in vivo cycle and its implications for muscle performance. J Exp Biol.

[CR5] Askew GN, Marsh RL, Ellington CP (2001). The mechanical power output of the flight muscles of blue-breasted quail (Coturnix chinensis) during take- off. J Exp Biol.

[CR6] Askew GN, Marsh RL (2002). Muscle designed for maximum short-term power output. J Exp Biol.

[CR7] Baylor SM, Hollingworth S (2003). Sarcoplasmic reticulum calcium release compared in slow-twitch and fast-twitch fibres of mouse muscle. J Physiol.

[CR8] Bhasin S, Woodhouse L, Storer TW (2001). Proof of the effect of testosterone on skeletal muscle. J Endocrinol.

[CR9] Boesiger B (1992). Histologie, immunocytologie, histochimie et innervation des fibres musculaires du muscle pectoralis major et du muscle supracoracoideus de Excalfactoria chinensis (L.). Acta Anat.

[CR10] Elemans CPH, Spierts ILY, Muller UK, van Leeuwen JL, Goller F (2004). Superfast muscles control dove’s trill. Nature.

[CR11] Ellerby DJ, Askew GN (2007). Modulation of pectoralis muscle function in budgerigars *Melopsittacus undulatus*. J Exp Biol.

[CR12] Ellington CP (1991). The novel aerodynamics of insect flight: applications to micro-air vehicles. J Exp Biol.

[CR13] Girgenrath M, Marsh RL (1997). *In vivo* performance of trunk muscles in tree frogs during calling. J Exp Biol.

[CR14] Girgenrath M, Marsh RL (1999). Power output of sound-producing muscles in the tree frogs *Hyla versicolor* and *Hyla chrysoscelis*. J Exp Biol.

[CR15] Girgenrath M, Marsh RL (2003). Season and testosterone affect contractile properties of fast calling muscles in the gray tree frog *Hyla chrysoscelis*. Am J Physiol.

[CR16] Hill AV (1950). The dimensions of animals and their muscular dynamics. Sci Prog.

[CR18] Johnston IA (1985). Sustained force development: specializations and variation among the vertebrates. J Exp Biol.

[CR19] Linari M, Dobbie I, Reconditi M, Koubassova N, Irving M, Piazzesi G, Lombardi V (1998). The stiffness of skeletal muscle in isometric contraction and rigor: the fraction of myosin heads bound to actin. Biophys J.

[CR20] Lowi-Merri TM, Benson RBJ, Claramunt S, Evans DC (2021). The relationship between variation and mode of locomotion in birds. BMC Biol.

[CR21] Marsh RL, Bennett AF (1986). Thermal dependence of contractile properties of skeletal muscle from the lizard *Sceloporus occidentalis* with comments on methods for fitting and comparing force-velocity curves. J Exp Biol.

[CR22] Marsh RL (1988). Ontogenesis of contractile properties of skeletal muscle and sprint performance in the lizard *Dipsosaurus dorsalis*. J Exp Biol.

[CR23] Marsh RL (1994). Jumping ability of anuran amphibians. Adv Vet Sci Comp Med.

[CR24] Marsh RL (1999). Contractile properties of muscles used in sound production and locomotion in two species of gray tree frog. J Exp Biol.

[CR25] Marsh RL, Taigen TL (1987). Properties enhancing aerobic capacity of calling muscles in gray tree frogs Hyla versicolor. Am J Physiol.

[CR26] Martin JH, Bagby RM (1981). Properties of rattlesnake shaker muscle. J Exp Zool.

[CR27] McLister JD, Stevens ED, Bogart JP (1995). Comparative contractile dynamics of calling and locomotor muscles in three hylid frogs. J Exp Biol.

[CR28] Mead AF, Osinalde N, Ortenblad N, Nielsen J, Brewer J, Vellema M, Adam I, Scharff C, Song Y, Frandsen U, Blagoev B, Kratchmarova I, Elemans CPH (2017) Fundamental constraints in synchronous muscle limit superfast motor control in vertebrates. eLife e29425. 10.7554/eLife.29425 PMC5699865 29165242

[CR29] Medler S (2002). Comparative trends in shortening velocity and force production in skeletal muscles. Am J Physiol.

[CR30] Moon BR, Hopp JJ, Conley KE (2002). Mechanical trade-offs explain how performance increases without increasing cost in rattlesnake shaker muscle. J Exp Biol.

[CR31] Pennycuick CJ, Rezende MA (1984). The specific power output of aerobic muscle, related to the power density of mitochondria. J Exp Biol.

[CR32] Peplowski MM, Marsh RL (1997). Work and power output in the hindlimb muscles of cuban tree frogs *Osteopilus septentrionalis* during jumping. J Exp Biol.

[CR33] Rome LC, Funke RP, McNeill Alexander R, Lutz G, Aldridge H, Scott F, Freadman M (1988). Why animals have different muscle fiber types. Nature.

[CR34] Rome LC, Lindsted S (1998). The quest for speed: muscles built for high-frequency contractions. News Physiol Sci.

[CR35] Rome LC, Cook. C, Syme DA, Connaughton MA, Ashely-Ross M, Klimov A, Tikunov B, Goldman YE (1999) Trading force for speed: why superfast crossbridge kinetics leads to super low forces. Proceedings of the National Academy of Sciences 96, 5826–5831 10.1073/pnas.96.10.5826 PMC21945 10318969

[CR36] Rome LC (2006). Design and function of superfast muscles: new insights into the physiology of skeletal muscle. Annu Rev Physiol.

[CR37] Schoenfeld BJ (2010). The mechanisms of muscle hypertrophy and their application to resistance training. J Strength Conditioning Res.

[CR38] Syme DA, Josephson RK (2002). How to build fast muscles: synchronous and asynchronous designs. Integr Comp Biol.

[CR39] Weis-Fogh T, Alexander RMcN, Pedley TJ (1977). The sustained power output from striated muscle. Scale Effects in Animal Locomotion.

[CR40] Woledge RC (1968). The energetics of tortoise muscle. J Physiol.

[CR41] Woledge RC, Curtin NA, Homsher E (1985). Energetic aspects of muscle contraction.

