# Supplementary material for: Adaptations for extremely high muscular power output: why do muscles that operate at intermediate cycle frequencies generate the highest powers?
Source: J Muscle Res Cell Motil. 2023 Jan 11;44(2):107–14. doi: 10.1007/s10974-022-09640-2 (PMC10329623; doi:10.1007/s10974-022-09640-2)
Supplement: Supplementary file 1 — Supplementary Material 1 [file 10974_2022_9640_MOESM1_ESM.pdf]

References

Adam, I., Maxwell, A., Rossler, H., Hansen, E.B., Vellema, M., Brewer, J. and Elemans, C.P.H. (2021). One-to-one innervation of vocal muscles allows precise control of birdsong. Current Biology 31, 3115-3124.

Altringham, J.D. and Johnston, I.A. (1988). The mechanical properties of polyneuronally innervated, myotomal muscle fibres isolated from a teleost fish (*Myoxocephalus scorpius*). Pflügers Arch 412:524–529.

Askew, G.N. and Marsh, R.L. (1998). Optimal shortening velocity (V/V_{max}) of skeletal muscle during cyclical contractions: length–force effects and velocity-dependent activation and deactivation. Journal of Experimental Biology 201, 1527-1540.

Askew, G.N. and Marsh, R.L. (2001). The mechanical power output of the pectoralis muscle of blue-breasted quail (*Coturnix chinensis*): the *in vivo* cycle and its implications for muscle performance. Journal of Experimental Biology 204, 3587-3600.

Boesiger, B. (1992). Histologie, immunocytologie, histochimie et innervation des fibres musculaires du muscle pectoralis major et du muscle supracoracoideus de *Excalfactoria chinensis* (L.). Acta Anatomica 145, 35-43.

Bone, Q., Johnston, I.A, Pulsford, A and Ryan, K.P. (1985). Contractile properties and ultrastructure of three types of muscle fibre in the dogfish myotome. Journal of Muscle Research & Cell Motility 7, 47–56.

Curtin, N.A. and Woledge, R.C. (1988). Power output and force-velocity relationship of live fibres from white myotomal muscle of the dogfish, *Scyliorhinus canicula*. Journal of Experimental Biology 140, 187-197.

Curtin, N.A. and Woledge, R.C. (1993a). Efficiency of energy conversion during sinusoidal movement of white muscle fibres from the dogfish *Scyliorhinus canicula*. Journal of Experimental Biology 183, 137–147.

Curtin, N.A. and Woledge, R.C. (1993b). Efficiency of energy conversion during sinusoidal movement of red muscle fibres from the dogfish *Scyliorhinus canicula*. Journal of Experimental Biology 185, 195–206.

Girgenrath, M. and Marsh, R.L. (1997). *In vivo* performance of trunk muscles in tree frogs during calling. Journal of Experimental Biology 200, 3101-3108.

Girgenrath, M. and Marsh, R.L. (1999). Power output of sound-producing muscles in the tree frogs *Hyla versicolor* and *Hyla chrysoscelis*. Journal of Experimental Biology 202, 3225-3237.

Gleeson, T.T., Nicol, C.J.M. and Johnston, I.A. (1984). Capillarization, mitochondrial densities, oxygen diffusion distances and innervation of red and white muscle of the lizard *Dipsosaurus dorsalis*. Cell Tissue Research 237, 253-258.

Guderley, H. and St-Pierre, J. (2002). Going with the flow or life in the fast lane: contrasting mitochondrial responses to thermal change. Journal of Experimental Biology 205, 2237-2249.

Hammond, L.C., Altrigham, J.D. and Wardles, C.S. (1998). Myotomal slow muscle function of rainbow trout *Oncorhynchus mykiss* during steady swimming. Journal of Experimental Biology 201, 1659-1671.

James, R.S., Cole, N.J., Davies, M.L.F. and Johnston, I.A. (1998). Scaling of intrinstic contractile properties and myofibrillar protein composition of fast muscle in the fish *Myoxocephalus scorpius* L. Journal of Experimental Biology 201, 901-912.

Johnston, I.A. (1985). Sustained force development: specializations and variation among the vertebrates. Journal of Experimental Biology 115, 239-251.

Josephson, R.K. (1984). Contraction dynamics of flight and stridulatory muscles of tettigoniid insects. Journal of Experimental Biology 108, 77-96.

Josephson, R.K. (1985a). Mechanical power output from striated muscle during cyclic contraction. Journal of Experimental Biology 114, 493–512.

Josephson, R.K. (1985b). The mechanical power output of a tettigoniid wing muscle during singing and flight. Journal of Experimental Biology 117, 357–368.

Kaiser, C.E. and George, J.C. (1973). Interrelationship amongst the avian orders Galliformes, Columbiformes, and Anseriformes as evinced by the fiber types in the pectoralis muscles. Canadian Journal of Zoology 51, 887-892.

Kiessling, K.H. (1977). Muscle structure and function in the goose, quail, pheasant, guinea hen, and chicken. Comparative Physiology and Biochemistry 57B, 287-292.

Kissane, R.W.P., Egginton, S. and Askew, G.N. (1998). Regional variation in the mechanical properties and fibre-type composition of the rat extensor digitorum longus muscle. Experimental Physiology 103, 111-124.

Lou, F., Curtin, N.A and Woledge, R.C. (2002). Isometric and isovelocity contractile performance of red muscle fibres from the dogfish *Scyliorhinus canicula*. Journal of Experimental Biology 205, 1585-1595.

Luff, A.R. and Atwood, H.L. (1971). Changes in the sarcoplasmic reticulum and transverse tubular system of fast and slow muscles of the mouse during postnatal development. The Journal of Cell Biology 51, 369-383.

Malamud, J. G. (1989). The tension in a locust muscle at varied muscle lengths. Journal of Experimental Biology 144, 479–494.

Marsh R.L. and Bennett, A.F. (1985). Thermal dependence of isotonic contractile properties of skeletal muscle and sprint performance of the lizard *Dipsosaurus dorsalis*. Journal of Comparative Physiology B 155, 541-551.

Marsh R.L. and Bennett, A.F. (1986). Thermal dependence of contractile properties of skeletal muscle from the lizard *Sceloporus occidentalis* with comments on methods for fitting and comparing force-velocity curves. Journal of Experimental Biology 126, 63-77.

Marsh R.L. and Olson, J.M. (1994). Power output of scallop adductor muscle during contractions replicating the *in vivo* mechanical cycle. Journal of Experimental Biology 193, 139-156.

Marsh, R.L. (1999). Contractile properties of muscles used in sound production and locomotion in two species of gray tree frog. Journal of Experimental Biology 202, 3215-3223.

Marsh, R.L. and Taigen, T.L. (1987). Properties enhancing aerobic capacity of calling muscles in gray tree frogs *Hyla versicolor*. American Journal of Physiology 252, R786-R793.

Martin, J.H. and Bagby, R.M. (1981). Properties of rattlesnake shaker muscle. Journal of Experimental Zoology 185, 293-300.

Mead, A.F., Osinalde, N., Ortenblad, N., Nielsen, J., Brewer, J., Vellema, M., Adam, I., Scharff, C., Song, Y., Frandsen, U., Blagoev, B., Kratchmarova, I. and Elemans, C.P.H. (2017). Fundamental constraints in synchronous muscle limit superfast motor control in vertebrates. eLife e29425.

Mizisin, A.P. and Josephson, R.K. (1987). Mechanical power output of locust muscle. Journal of Comparative Physiology A 160, 413–419.

Olson, J.M. and Marsh, R.L. (1993). Contractile properties of the striated adductor muscle in the bay scallop *Argopecten irradians* at several temperatures. Journal of Experimental Biology 176, 175-193.

Park-Holohan, S.-J., West, T. G., Woledge, R. C., Ferenczi, M. A., Barclay, C. J. and Curtin, N.A. (2010). Effect of phosphate and temperature on force exerted by white muscle fibres from dogfish. Journal of Muscle Research and Cell Motility 31, 35–44.

Phillip, E.E.R., Schmidt, M., Gsottbauer, C., Sanger, A.M. and Abele, D. (2008). Size- and age-dependent changes in adductor muscle swimming physiology of the scallop *Aequipecten opercularis*. Journal of Experimental Biology 211, 2492-2501.

Rome, L.C., Cook., C., Syme, D.A., Connaughton, M.A., Ashely-Ross, M., Klimov, A., Tikunov, B. and Goldman, Y.E. (1999). Trading force for speed: why superfast crossbridge kinetics leads to super low forces. Proceedings of the National Academy of Sciences 96, 5826-5831.

Rosser, B.W.C, George, J.C. and Frombach, S.K. (1987). Architecture of the pectoralis muscle of the Japanese quail (*Coturnix japonica*): histochemical and ultrastructural characterization, and distribution of muscle fiber types. Canadian Journal of Zoology 65, 63-71.

Rome, L.C., Syme, D.A., Hollingworth, S., Lindstedt, S.L. and Baylor, S.M. (1996). The histle and the rattle: the design of sound producing muscles. Proceedings of the National Academy of Sciences 93, 8095-8100.

Sanger, J.W. and Sanger, J.M. (1985). Sarcoplasmic reticulum in the adductor muscles of a Bermuda scallop: comparison of smooth versus cross-striated portions. Biological Bulletin 168, 447-460.

Schaeffer, P.J., Nichols, S.D. and Lindstedt, S.L. (2007). Chronic electrical stimulation drives mitochondrial biogenesis in skeletal muscle of a lizard, *Varanus exanthematicus*. Journal of Experimental Biology 210, 3356-3360.

Swoap, S.J., Johnson, T.P., Josephson, R.K. and Bennett, A.F. (1993). Temperature, muscle power output and limitations on burst locomotor performance of the lizard *Dipsosaurus dorsalis*. Journal of Experimental Biology 174, 185–197.

Swoap, S. J., Caiozzo, V. J. and Baldwin, K. M. (1997). Optimal shortening velocities for *in situ* power production of rat soleus and plantaris muscles. American Journal of Physiology 273, C1057-C1063.

Wakeling, J.M. and Johnston, I.A. (1998). Muscle power output limits fast-start performance in fish. Journal of Experimental Biology 201, 1505–1526.

West, T.G., Donohoe, P.H., Staples, J.F. and Askew, G.N. (1998). Tribute to R. G. Boutilier: The role for skeletal muscle in the hypoxia-induced hypometabolic responses of submerged frogs. Journal of Experimental Biology 209, 1159–1168.

Young, I.S. and Rome, L.C. (2001). Mutually exclusive muscle designs: the power output of the locomotory and sonic muscles of the oyster toadfish (*Opsanus tau*). Proceedings of the Royal Society B 268, 1965-1970.
