# Supplementary material for: Adaptations for extremely high muscular power output: why do muscles that operate at intermediate cycle frequencies generate the highest powers?
Source: J Muscle Res Cell Motil. 2023 Jan 11;44(2):107–14. doi: 10.1007/s10974-022-09640-2 (PMC10329623; doi:10.1007/s10974-022-09640-2)
Supplement: Supplementary file 2 — Supplementary Material 2 [file 10974_2022_9640_MOESM2_ESM.pdf]

Animal	Muscle	Twitch rise (ms)	P0 (kN m-2)	Frequency in vivo	Power (W/kg)	Vmax	Myobrils (% Vol)	Mitochondria (% Vol)	SR (% Vol)	Other (%)	Myofibrillar stress	Myofibrillar power	Work (J/kg)	Myofibrillar Work (	Cycle Duration (ms	Paper & Notes
Toadfish	red	142	192	0.8	4.72	2.43	70	30			274.2857143	6.742857143	5.9	8.428571429	1250	Rome et al., 1999 for stress - note data inconsistent between table and text; Mead et al for relative volumes; opt freq taken from Young & Rome; mito vol reported in ref 30 in Rome et al 1999
Mouse	soleus	16.2	269	3.3	51.3		81	6	4		332.0987654	63.33333333	15.54545455	19.19191919	303.030303	Askew and Marsh, 1998; %SR from Luff & Atwood, 1971
Dogfish	red		142.4	1.02		1.814	62.2	21.3	2	1.53	228.9389068				980.3921569	Lou et al 2002; rel vols Bone et al 1986; Curtin & Woledge 1993b only relative power reported
Sculpin (Myoxocephalus scorpius)	red			2	8		68	20				11.76470588	4	5.882352941	500	Altringham & Johnson, 1988
Trout	red	119	139	2	20		60.3	27		7.5	230.5140962	33.16749585	10	16.58374793	500	Hammond et al 1998; Guderley & St Pierre 2002 for myo vol taken for fish acclimated at 16 deg C
Animal	Muscle	Twitch rise (ms)	P0 (kN m-2)	Frequency in vivo	Power (W/kg)	Vmax	Myobrils (% Vol)	Mitochondria (% Vol)	SR (% Vol)	Other (%)	Myofibrillar stress	Myofibrillar power	Work (J/kg)	Myofibrillar Work (J/kg)		
Rat	EDL	9.14	300	15	85	14.8		8.8					5.666666667		66.66666667	Kissane et al., 2018 - data for lateral compartment
Toadfish	white	66.7	244	4	27.64	4.12	85	1	10	4	287.0588235	32.51764706	6.91	8.129411765	250	Rome et al., 1999 for stress - note data inconsistent wbetween table and text; Mead et al 2017 for relative volumes; opt freq taken from Young & Rome, 2001; mito vol reported in ref 30 in Rome et al 1999
scallop	adductor	135.2	241.7	1.82	29.1	5.35	95.04	1.0	4		254.3139731	30.61868687	15.98901099	16.82345432	549.4505495	Marsh & Olson, 1994; Olson & Marsh, 1993; %SR in Sanger & Sanger, 1985; %Mitos Phillip et al 2008
Scorpaena notata	rostral fast	9.32	239	10.9	142.73								13.09449541		91.74311927	Wakeling & Johnston (1998)
Mouse	EDL	7.3	243	8.8	132.2		75	8	6		324	176.2666667	15.02272727	20.03030303	113.6363636	Askew and Marsh, 1998; %SR from Luff & Atwood, 1971
Rana temporaria	sartorius		198.8	2	13.2								6.6		500	West et al., 2006
Dipsosaurus	iliofibularis (white region)	11.1	214	20.1	153.7	18.7	86.2	3.8	10		248.2598608	178.3062645	7.646766169	8.870958433	49.75124378	Swoap et al 1993; Marsh & Bennett (1985) for Vmax at 40; Gleeson et al 1984 mito density; Schaeffer et al 2007 for SR (NB diff species but similar mito density)
Blue breasted quail	pectoralis	10.8	205	23.2	349.1	32	75	19.8	5.2		273.3333333	465.4666667	15.04741379	20.06321839	43.10344828	Askew and Marsh 2001; stress from Johnston 1985 as Askew and Marsh did not measure at optimal length; muscle composition - Relative No from Boesiger 1992; Relative fibre type area calc using fibre size in Kiessling 1977; composition estimated from Rosser et al 1987 (figs 4b and 5b) and overall composition determined from relative fibre composition by area; calc % area agrees with Kaiser & George, 1973
Rat	Plantaris		293	6	140.4								23.4		166.6666667	Swoap et al (1997)
Neoconocephalus triops	metathoracic (flight)	7.8	126	25	61	13.6	57				221.0526316	107.0175439	2.44	4.280701754	40	at 30 deg C; Josephson, 1984, 1985a & b
Locust (S gregaria)	Tcx2 (flight & walking)	17	32	20	75	5.8	55	26	18.15		58.18181818	136.3636364	3.75	6.818181818	50	Stress & twitch time from Malamud 1989; power from Mizisin & Josephson, 1987
Dogfish	white		241	3.5	46.9	4.5	77.8	5.4	5.8		309.7686375	60.28277635	13.4	17.22365039	285.7142857	Curtin & Woledge (1993a); stress Park-Holohan et al., 2010; rel vols Bone et al 1986; stress Curtin & Woledge 1988 (NB slightly higher value in Lou et al 2002)
Sculpin (Myoxocephalus scorpius)	white	32.95	281	5	27	4.75	86.3		13.7		325.608343	31.28621089	5.4	6.257242178	200	Altringham & Johnston 1988; SR vol from trout; twitch time calculated from James et al 1998 (26 cm fish)
Animal	Muscle	Twitch rise (ms)	P0 (kN m-2)	Frequency in vivo	Power (W/kg)	Vmax	Myobrils (% Vol)	Mitochondria (% Vol)	SR (% Vol)	Other (%)	Myofibrillar stress	Myofibrillar power	Work (J/kg)	Myofibrillar Work (J/kg)		
Toadfish	swimbladder	7	24	200	14.19	11.8	69	4	24	3	55	20.56521739	0.07095	0.102826087	5	Rome et al., 1999 for stress - note data inconsistent between table and text; Mead et al 2017 for relative volumes; opt freq taken from Young & Rome, 2001; mito vol reported in ref 30 in Rome et al 1999
Rattlesnake	shaker	5.68	29.4	90		7.68	32	26	26	16	91.875				11.11111111	Rome et al., 1996; Mead et al 2017; stress from Martin & Bagby, 1973
Zebrafinch	syrinx	3.5	5.97	225	6.2		50	22	15	13	11.94	12.4	0.027555556	0.055111111	4.444444444	Mead et al 2017 twitch rise time & power est. from Elemans et al 2008 PLoS One; Stress from Adam et al., 2021
Hyla versicolor	external obliques	27	99	21	58	9.13	55	19.5		14	180	105.4545455	2.761904762	5.021645022	47.61904762	Marsh & Taigen , 1987 for organelle vol; Girgenrath & Marsh 1997, 1999 for power & strain; Marsh 1999 for Vmax & twitch rise
Hyla chrysoscelis	external obliques	16	74.5	44	54	10.54	55	19.5			135.4545455	98.18181818	1.227272727	2.231404959	22.72727273	Girgenrath & Marsh, 1997, 1999; Marsh 1999 for Vmax and twitch rise; organelle vol not available taken from versicolor
Neoconocephalus triops	mesothoracis (stridulation & flight)	4.2	58	100	17.9	10.2	44				131.8181818	40.68181818	0.179	0.406818182	10	at 30 deg C; Josephson, 1984, 1985a & b
